# Minimizing Iridium Oxide Electrodes for High Visual Acuity Subretinal Stimulation

**DOI:** 10.1523/ENEURO.0506-20.2021

**Published:** 2021-12-10

**Authors:** Samir Damle, Maya Carleton, Theodoros Kapogianis, Shaurya Arya, Melina Cavichini-Corderio, William R. Freeman, Yu-Hwa Lo, Nicholas W. Oesch

**Affiliations:** 1 Department of Bioengineering, University of California San Diego, La Jolla, CA 92093; 2 Department of Psychology, University of California San Diego, La Jolla, CA 92093; 3 Department of Electrical and Computer Engineering, University of California San Diego, La Jolla, CA 92161; 4 Jacobs Retina Center at Shiley Eye Institute, Department of Ophthalmology, University of California San Diego, La Jolla, CA 92093

**Keywords:** blindness, electrical stimulation, neural stimulation, prosthetic, retina, retinal prosthetic

## Abstract

Vision loss from diseases of the outer retina, such as age-related macular degeneration, is among the leading causes of irreversible blindness in the world today. The goal of retinal prosthetics is to replace the photo-sensing function of photoreceptors lost in these diseases with optoelectronic hardware to electrically stimulate patterns of retinal activity corresponding to vision. To enable high-resolution retinal prosthetics, the scale of stimulating electrodes must be significantly decreased from current designs; however, this reduces the amount of stimulating current that can be delivered. The efficacy of subretinal stimulation at electrode sizes suitable for high visual acuity retinal prosthesis are not well understood, particularly within the safe charge injection limits of electrode materials. Here, we measure retinal ganglion cell (RGC) responses in a mouse model of blindness to evaluate the stimulation efficacy of 10, 20, and 30 μm diameter iridium oxide electrodes within the electrode charge injection limits, focusing on measures of charge threshold and dynamic range. Stimulation thresholds were lower for smaller electrodes, but larger electrodes could elicit a greater dynamic range of spikes and recruited more ganglion cells within charge injection limits. These findings suggest a practical lower limit for planar electrode size and indicate strategies for maximizing stimulation thresholds and dynamic range.

## Significance Statement

Neural prosthetics offer hope to cure intractable neurologic disorders. To enable fine control over patterns of neural activity, stimulating electrode size must be decreased to the scale of neurons. We examined how electrode size at this scale influences stimulation threshold and the range of possible responses, by fabricating planar iridium oxide electrodes between 10 and 30 μm in diameter, and examined neural stimulation in a mouse model of retinal degeneration. This work provides new insights into how small stimulation electrodes translate charge into neural activity and the physical factors that contribute to differences between responses over this range of electrode sizes. This has important implications for the design of high-acuity retinal prosthetics, as well as next-generation neural stimulators.

## Introduction

The number of patients with sight-threatening retinal diseases is increasing (Wong et al., 2014; Bourne et al., 2017). Dystrophic or degenerative retinal diseases result in the progressive loss of photoreceptors, causing vision loss (Hartong et al., 2006; Jones et al., 2016; Pennington and DeAngelis, 2016). Currently there are limited options to slow progression, and no options to recover lost vision. Early retinal prostheses have demonstrated promising prosthetic visual acuity results between 20/500 and 20/1520 in clinical trials (Humayun et al., 2012; Stingl et al., 2015; Palanker et al., 2020). To improve on the visual benefits provided by retinal prostheses, recent research efforts have focused on developing implants with more densely packed microelectrodes to support higher visual acuity (Lorach et al., 2015; Ha et al., 2016; Damle et al., 2020).

While different retinal implant technologies have been developed to translate an image into a spatial pattern of electrical stimulation, all deliver electric charge through electrodes to stimulate action potentials in retinal ganglion cells (RGCs), the output neurons of the retina. RGCs can be directly activated by electrical stimulation or indirectly through synaptic input when upstream neurons, such as retinal bipolar cells are excited. The ultimate goal of retinal prosthetics is to create patterns of action potentials in the RGC layer that encode the spatiotemporal properties of visual scenes, which are then relayed to the brain (Goetz and Palanker, 2016; Yue et al., 2016).

The limit of prosthetic spatial resolution is determined by the size and spacing of stimulating electrodes in implanted arrays (Palanker et al., 2005; Wilke et al., 2011). To increase spatial resolution, there is significant pressure to decrease electrode size; however, there is a trade-off between electrode size and the amount of charge that can be safely injected, termed the charge injection capacity (CIC; Ganji et al., 2017). Beyond this limit, irreversible damage to both neural tissue and electrode materials occur (Cogan, 2008). Retinal implants currently in clinical testing use planar disk shaped electrodes, ranging from 30 to 200 μm diameters and are composed of sputtered iridium oxide film (SIROF), an electrode material with high CIC (Cogan et al., 2009; Shire et al., 2009; Boinagrov et al., 2016; Ha et al., 2016; Yang et al., 2016).

Electrical stimulation of retinal cells has been studied extensively with *ex vivo* retinal preparations using a wide variety of electrode materials and geometry (Stett et al., 2000; Yang et al., 2011, 2018; Cai et al., 2013; Sim et al., 2014; Im and Fried, 2015; Stutzki et al., 2016). These studies have provided valuable insight into electrical requirements for some electrode configurations; however, there is still considerable variability in the results reported, and much less attention to the range of possible responses beyond the stimulation threshold, particularly within the practical limits of safe injectable charge. As a result, there is little consensus of ground truth principals that establish a framework for electrode development for high visual acuity subretinal prostheses.

Here, we address these questions by examining retinal stimulation elicited by planar microelectrodes of 10, 20, and 30 μm diameter. We first established the charge limits of these electrodes, to establish the realistic bounds of maximal stimulation. We then varied current magnitude and pulse width within these limits to characterize the stimulation threshold and dynamic range of RGC spiking at each electrode diameter, both important measures of efficacy. We found that smaller electrodes have lower charge thresholds, but recruited fewer RGS and have a reduced dynamic range compared with larger electrodes. Importantly, these results demonstrate that charge density alone does not normalize efficacy of stimulation across different electrode diameters, but electric field modeling shows that the spatial extent of the electric field may be important when determining stimulation efficiency even at the same charge density. Since electric field size is influenced by both electrode size and stimulation charge, this finding places constraints on neural prosthetic electrode design. Taken together, this work establishes a framework to develop and compare new microelectrodes for high visual acuity prosthesis within realistic limits.

## Materials and Methods

### Microelectrode fabrication

**Figure 1.**
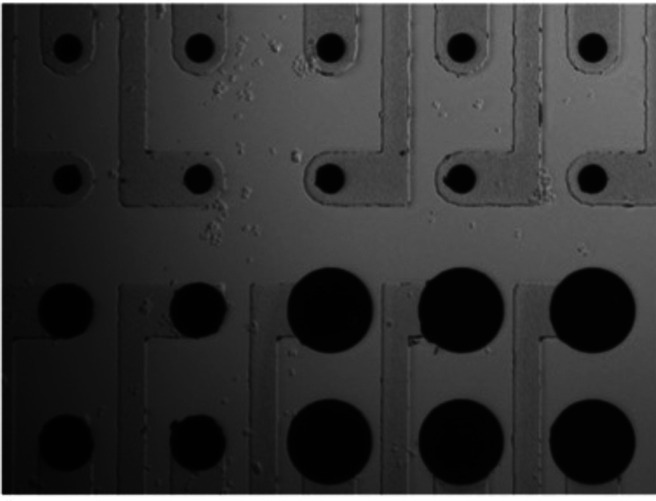
Image of a region of the microfabricated array of sputtered iridium oxide electrodes of 10 μm (top), 20 μm (bottom left), and 30 μm (bottom right) diameter electrodes at 50 μm pitch.

Iridium oxide electrodes were patterned by photolithography. Transparent conductive traces of indium tin oxide (ITO) were deposited on borosilicate glass by RF magnetron sputtering and were insulated by a 200 nm layer of SiNx deposited by plasma-enhanced chemical vapor deposition (PECVD). Iridium oxide electrodes of 10, 20, and 30 μm diameter stimulating electrodes and 400-μm return electrodes were formed by reactive DC sputtering of iridium metal in an argon (90%) and oxygen (10%) gas mixture at a thickness of 600 nm. Electrodes were spaced 50 μm apart, from electrode center to center. Electrode arrays consisted of four rows of eight electrodes, with the top two rows consisting of 10 μm diameter electrodes, the bottom left quadrant of electrodes consisting of 20 μm diameter electrodes, and the bottom right quadrant consisting of 30 μm diameter electrode. (Fig. 1). Finally, a 1.5 μm insulating layer of parylene-C was deposited over the electrode array (PDS 2010 Coater) and iridium oxide electrodes were exposed by oxygen plasma reactive ion etching (RIE) etching (Oxford Plasmalab 80; Fig. 1)

### Electrochemical characterization

Electrochemical characterization was performed using a three-electrode configuration consisting of the iridium oxide electrode as the working electrode, an Ag/AgCl electrode as the reference, and a platinum wire as the counter electrode (Gamry Interface 1000E). Cyclic voltammetry measurements were performed to determine the charge storage capacity (CSC) of the electrodes. The electrode potential was swept cyclically between −0.6 and 0.8 V, relative to the reference electrode at constant scan rate of 200 mV/s at 10 mV increments while measuring the flow of current from the working electrode to the counter electrode. The CSC was calculated using the time integral of the cathodic and anodic currents up to the potential limits of the water window; the voltage range where electrochemical reactions occurring at the electrode-electrolyte interface are reversible.

The CIC of the electrode, defined as the maximum amount of charge that can be delivered without polarizing the electrode potential beyond the water window, was determined by measuring the transient voltage change between the working and reference electrode in response to a square biphasic anodic first current pulse injected between the working and counter electrode, measured as the near-instantaneous voltage change immediately after the current pulse is terminated (E_ma_ or E_mc_ = ΔV – V_a_; Cogan, 2008). The electrode polarization at the anodal and cathodal phase were measured 10 μS after the end of each respective phase of the stimulation pulse. All measurements were made in 126 mm NaCl.

### Retina explant and loose patch electrical recording

All experimental methods and animal care procedures were conducted in accordance with National Institutes of Health guidelines and were approved by the University of California, San Diego Institutional Animal Care and Use Committee. Adult rd1 or rd10 mice, of either sex (> postnatal day 60) with photoreceptor cell degeneration were anesthetized with isoflurane and euthanized by decapitation and their retinas were isolated and maintained in Ames medium oxygenated and equilibrated with 95% O_2_, 5% CO_2_. Retina pieces, ∼2 × 2 mm, were transferred to a custom recording chamber and placed over stimulating electrodes on the bottom of the custom recording chamber, ganglion cell side up. The chamber was placed under an upright microscope and perfused with Ames solution (4 ml/min) at 35°C. Microscopy was used to visualize and confirm contact between the outer portion of the retina and the electrode array surface. RGCs were visualized and targeted using IR differential interference contrast video microscopy. Given that the stimulating electrode pitch is 50 μm, this means that our recordings are from cells that are within ∼25 μm of the stimulating electrodes. Furthermore, given our arrangement of the different electrode diameters on the same array, we have several regions, where we can record from cells that are equidistant between different electrode diameters.

Recording electrodes were pulled from borosilicate capillary glass to have a final resistance of 4–5 MΩ and filled with Ames medium. Loose-patch recordings were made from ganglion cells and action potentials were recorded in voltage-clamp mode using a Multiclamp 700b (Molecular Devices) patch-clamp amplifier. Signals were filtered at 4 kHz (4-pole Bessel), digitized at 20 kHz with an ITC-18 (HEKA Electronik) data acquisition board and saved to a PC for offline analysis using custom acquisition software in IgorPro 7 (WaveMetrics). SIROF electrons were wired to a 32-channel RHS2000 stim and recording system (Intan Technologies). Charge-balanced, anodic first, square biphasic current pulses were generated on the RHS2000, which was triggered by our acquisition software, and delivered to an individual stimulating electrode nearest to the cell of interest. Individual stimulus pulses or pulse trains were delivered every 10 s and repeated five times. Individual cell responses were calculated from the average of the five repeats. Spontaneous activity was subtracted from stimulation evoked activity by measuring the spontaneous firing rate for 2 s before the stimulus, and measuring any change in evoked response above this baseline level. To measure threshold, half-max and dynamic range, linear interpolation between stimulation levels was used to estimate the current needed to achieve a particular metric.

### Simulation of electric field

Simulations of electric field were performed using a 3-D finite element model of the electrode array and retina for static current flow inside of a volume conductor using COMSOL Multiphysics 5.2b (COMSOL AB) on a personal computer running Windows 7. A model of a single stimulating electrode was constructed for 10, 20, and 30 μm diameters. The electrode was centered inside of a passive domain consisting of a homogenously conductive (0.2 S/m) sphere of 18 mm diameter (Wang and Weiland, 2015). A single large return electrode of 200 μm diameter was positioned at a distance of 7.5 mm from the center of the electrode array.

The magnitude of the electric field (E) at a particular electrode-cell separation distance is a function of the current density produced at an electrode ( J) and the tissue conductivity (σ, assumed to be constant). The voltage potential distribution inside of the passive domain was modeled according to the Poisson equation and continuity equation (Wilke et al., 2011):

∇⋅J=∇⋅(−σ∇V)=0.

The electric field was determined from the voltage distribution in COMSOL by calculating the gradient of the voltage distribution:

E=−∇V.

The distribution of the electric field from the stimulating electrode into the 3-D volume was calculated for a fixed current density set at the surface of each electrode. This model allowed for a simplified electrostatic approach to understand the effect of stimulating current magnitude within safe CIC limits of each electrode diameter in terms of the magnitude and spatial distribution of the electric field available to induce stimulation of retinal neurons.

### Code accessibility

The code/software described in the paper is freely available online at https://github.com/noeschlab/noeschlab/tree/noeschlab-ElectrodeSizeCOMSOL. The code is available as COMSOL .mph files in Extended Data 1.

10.1523/ENEURO.0506-20.2021.ed1 Extended Data 1 The extended data consists of three COMSOL .mph files labeled 10-μm electrode 20-μm pitch.mph, 20-μm electrode 20-μm pitch.mph, and 30-μm electrode 20-μm pitch.mph. These files allow the user to view and rerun the COMSOL simulations used in this manuscript and described in the methods section. Download Extended Data 1, ZIP file.

## Results

### Electrochemistry

To examine how electrical stimulation of the retina is influenced by the electrode diameter we fabricated SIROF electrodes at 10, 20, and 30 μm diameters. SIROF is widely considered to be the state-of-the-art electrode material for neural prosthetics (Cogan, 2008). These diameters were chosen because they correspond to the size of electrodes that can be packed with an electrode pitch <50 μm, the necessary density for a retinal prosthetic array to reach the spatial acuity equivalent of legal blindness (Palanker et al., 2005). Our goal was to explore the range of neural responses that can be realistically stimulated within the electrochemical limits for electrodes of this scale.

**Figure 2.**
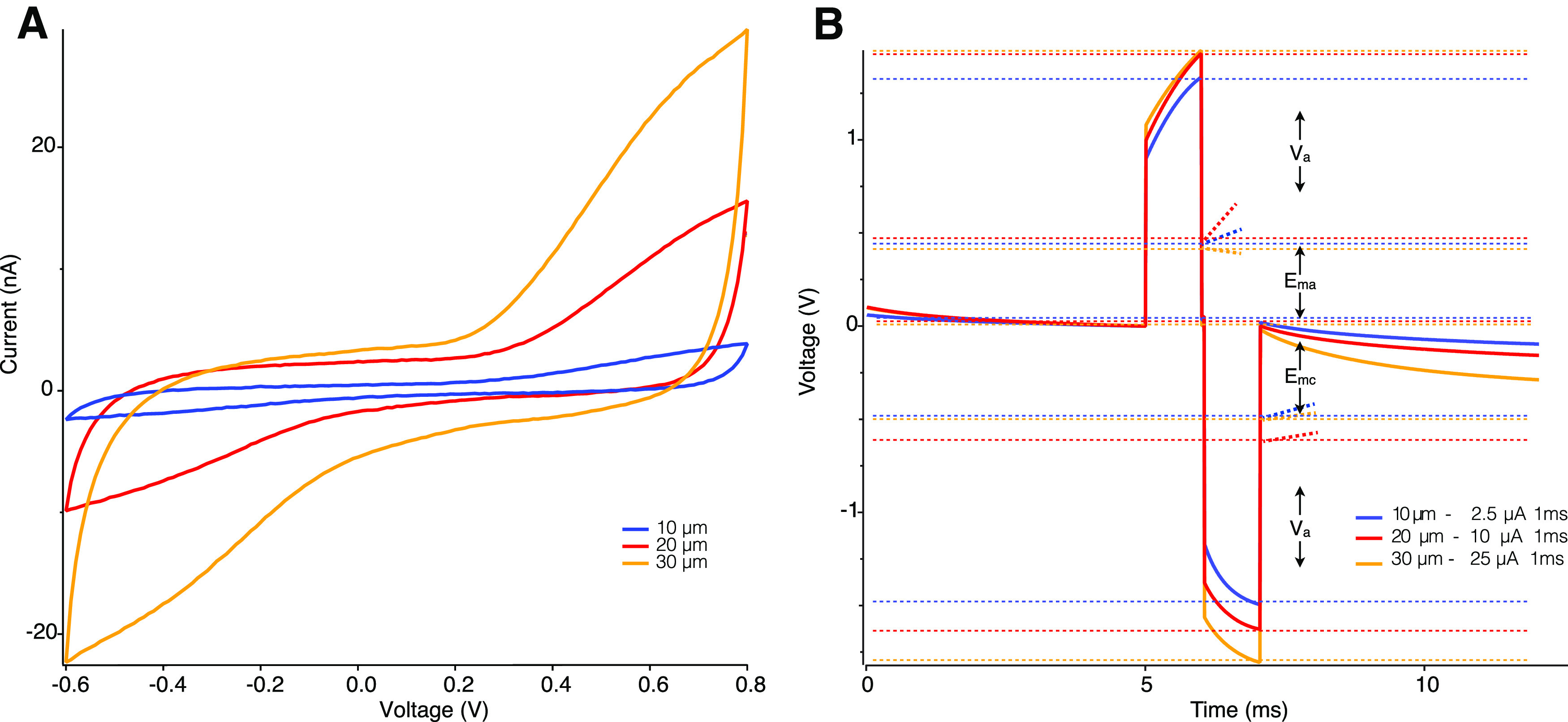
Electrochemical characterization of 10 μm (blue), 20 μm (red), and 30 μm (orange) sputtered iridium oxide electrodes. ***A***, Cyclic voltammetry scan used to calculate the electrochemical CSC of the electrodes. ***B***, Voltage transients in response to a biphasic current pulse used to calculate the safe CIC of electrodes within the polarization limits of the water window of iridium oxide. The rapid change in potential represents the voltage drop across the electrolyte solution (V_a_) while the gradual change in potential represents the polarization of the electrode (E_ma_ and E_mc_). Dashed lines represent the voltage boundaries for V_a_ and E_ma_ and E_mc_ for 10, 20, and 30 μm electrodes in blue, red, and orange, respectively.

We first characterized the CSC of the electrodes at each diameter to ensure their performance was consistent with those typically used in retinal prostheses. CSC provides information about the total amount of electroactive material on the electrode at near-equilibrium conditions. The magnitude of charge that each electrode can deliver scales with the electrode surface area as expected (Fig. 2*A*). The measured anodal and cathodal charge storage capacities of all electrode diameters were identical at 12 and 17 mC/cm^2^, respectively. These results fall within the range of previously reported studies of iridium oxide electrodes (Cogan et al., 2009).

Although it has become common practice to characterize electrodes by their CSC, it does not accurately reflect the amount of charge that can be safely delivered per phase during realistic neural stimulation conditions. This property, called the CIC (Cogan, 2008), imposes a limit on the maximum amount of charge that can be used for stimulation without driving the electrode polarization to a level at which irreversible reactions occur that could damage the electrode or tissue. The electrode polarization limit is known as the water window and occurs at −0.6V for cathodal stimulation and 0.8 V for anodal stimulation for SIROF electrodes. The measured charge injection limit of sputtered iridium oxide electrodes was 3.3 ± 0.2 mC/cm^2^, also within the range of previously reported studies of SIROF (Cogan, 2008; Cogan et al., 2009; Haas et al., 2020; Negi et al., 2012). The total safe injectable charge per stimulation pulse scales with area, equaling 2.5, 10, and 25 nC for 10, 20, and 30 μm electrodes, respectively (Fig. 2*B*).

### Stimulation evoked ganglion cell responses

To examine how the retina responds to stimulation by these electrodes we mounted the electrode arrays on the bottom of our standard electrophysiological recording chamber, and placed the retina outer side down, such that the inner nuclear layer was in contact with the electrode array on the bottom of the chamber and ganglion cells faced up. We used loose-patch recordings to measure ganglion cell spiking from whole mount RD1 or RD10 retina (ages 60–140 d). We did not observe a difference between RD1 and RD10 retinas across a range of stimulation intensities (ANOVA, *p* = 0.91) and grouped RD1 and RD10 results when both were used.

**Figure 3.**
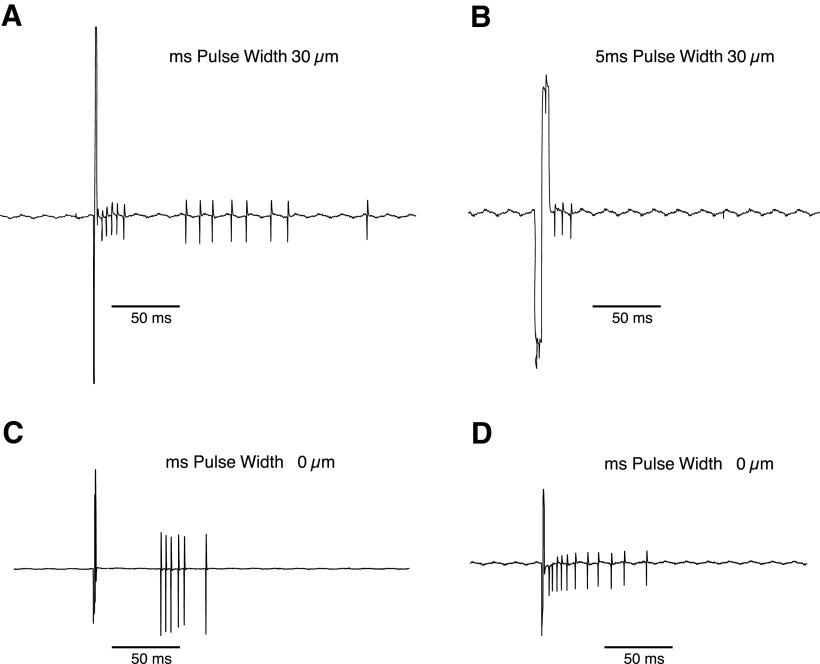
Example spiking responses of RGCs in response to subretinal electrical stimulation with SIROF electrodes measured by loose patch clamp. Stimulation with a 30 μm electrode at 1-ms (***A***) or 5-ms (***B***) pulse width. Stimulation with 1 ms pulse width for a (***C***) 20 μm electrode and (***D***) 10 μm electrode (***D***).

Given, that we image the electrode array and the RGCs simultaneously, we know the position and distance between the cell of interest and the stimulating electrode. We stimulated cells on the electrode nearest to the cell, when possible, meaning the stimulating electrode was typically 25 μm or less from the cell of interest, given our 50 μm pitch. We delivered anodic first, biphasic, charged balanced constant current pulses with 1 or 5 ms duration per phase and varied the magnitude of the stimulating current up to the CIC limit of the electrode. Anodic first pulses have previously been shown to have lower charge thresholds for subretinal stimulation (Boinagrov et al., 2014). Electrical stimulation elicited bursts of action potentials following charge delivery (Fig. 3).

### Maximizing retinal response within charge injection limits

Retinal stimulation is often described in terms of total charge delivered by a stimulation pulse. By definition, charge is the product of the magnitude of stimulating current and the duration of the stimulus pulse. Given the limits on safe injectable charge imposed by the electrochemical properties of the electrode, it is important to optimize the impact of current magnitude and pulse width for stimulation efficacy. In this work, we define efficacy as the ability to produce ganglion cell responses in response to electrical stimulation. It is commonly understood that there may be multiple aspects of ganglion cell responses that may be important for different aspects of prosthetic vision restoration. Here, we largely focus on two measurements of electrical stimulation, the stimulation threshold, defined as the current needed to elicit a spike on 50% of the trials, and the dynamic range, defined as the ratio of the maximal number of spikes evoked within the CIC limit and the spikes evoked at the threshold.

**Figure 4.**
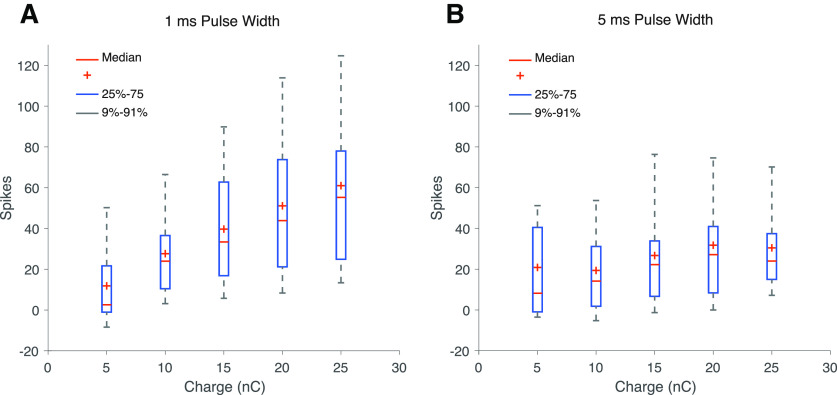
Average response of RGC to stimulation with a 30-μm diameter electrode using 1 or 5 ms pulse widths plotted against stimulating charge within 25 nC charge injection limits. ***A***, Range of spiking responses for stimuli with a 1 ms pulse width. ***B***, Range of spiking responses for stimuli with a 5 ms pulse width. For both pulse widths, average spikes increase with increasing charge. At the same charge level, shorter pulse widths elicit more spikes (for 1 ms, 5 to 20 nC range, *n* = 45; at 25 nC, *n* = 10; for 5 ms, *n* = 21). Red plus denote mean.

To examine how responses to the same amount of charge differ with different combinations of current and time we used a 30 μm diameter electrode to deliver an equivalent level of charge for a combination of stimulus current and duration (Fig. 4). We characterized spiking responses by measuring the total number of spikes elicited as well as the pattern of evoked spikes. The average magnitude of the spike response was significantly greater at each fixed stimulating charge level for a 1 versus a 5 ms pulse for all charge levels >5 nC (*t* test, *p* < 0.05; *n* = 45 for both pulse widths). There were nearly twice as many total spikes at the maximum stimulating charge of 25 nC (*t* test, *p* < 0.05); however, only a subset of cells was tested at this charge level out of concern for possibly damaging electrodes by operating at the upper limit of the safe injectable charge (*n* = 10 for 1 ms pulse width and *n* = 21 for 5 ms pulse width). As expected, the responses of RGCs increase with increasing charge for both pulse widths. Overall, for the entire range of safe injectable charge, the shorter 1 ms pulse width elicited a greater number of spikes than the longer 5 ms pulse width (ANOVA, *p* < 0.001).

**Figure 5.**
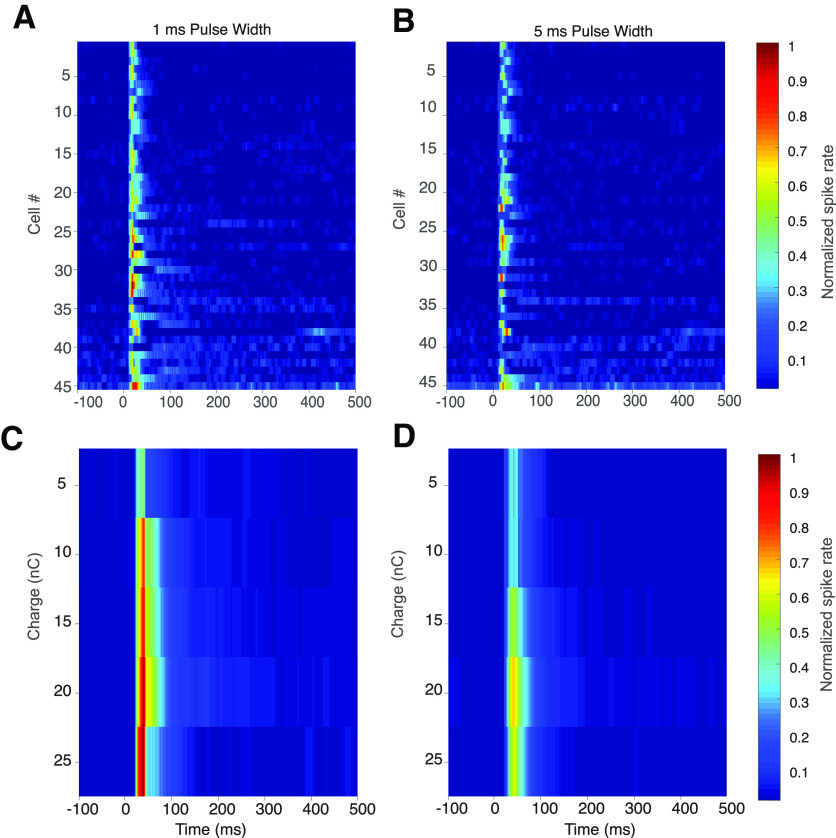
Heat map of normalized spike rates evoked by stimulation using 1 and 5 ms pulse width using a 30 μm iridium oxide electrode for 50 ms before stimulation to 500 ms after stimulation. Top row, Comparison of spike magnitude and latency for 45 RGCs stimulated with 20 nC of charge using either (***A***) 1 ms pulse or (***B***) 5 ms pulse widths. Cells are ordered by the magnitude of their response to 1 ms stimulation between time 0 and 500 ms. Bottom row, Average spike rates for a range of equivalent charge for 1 ms (***C***) and 5 ms (***D***) stimuli, for cells in ***A***, ***B***. Color scale is normalized spiking to maximal rate in dataset.

To examine differences in the pattern of spikes following stimulation between 1 and 5 ms pulse widths at equivalent charge, we plotted the peristimulus time histogram for spikes up to 500 ms after stimulation, across a population of 45 cells. In general, the pattern of evoked short and long latency spikes for both pulse widths appear similar within the same cell, but there is significant variation in spiking behavior between cells over the entire population of observed cells. This can be observed when evaluating the pattern of evoked responses at 20 nC for 1 and 5 ms pulse widths for all cells (Fig. 5*A*,*B*). Comparing the average response at each pulse width at every charge level, there is a greater magnitude of both short latency (<100 ms) and long latency spikes (<100 ms) for the 1 ms pulse duration (Fig. 5*C*,*D*).

### Influence of electrode diameter on neural response

High acuity retinal prostheses require an array with a high density of stimulating electrodes, because electrode spacing should be directly correlated with the resolution of restored vision (Palanker et al., 2005; Moghadam et al., 2013; Yang et al., 2016). Therefore, significant effort has been dedicated to decreasing stimulating electrode area and spacing, however, smaller electrodes have less available CIC. To examine how electrode diameter influences electrical excitation of the retinal circuitry, we compared the effectiveness of 10, 20, and 30 μm diameter SIROF electrodes at achieving retinal stimulation within the limits of their respective CIC. Based on the above results, showing that the maximum spike response within the CIC is larger for shorter pulses, we chose to use stimuli with a 1 ms pulse width for this comparison. Because repetitive trains of stimulation pulses may likely be used to encode temporal properties of vision in retinal prosthetics as opposed to single pulses in isolation, we also examined responses to pulse trains of 20 simulation pulses at 500 Hz (Lee et al., 2013; Im and Fried, 2016).

We were able to evoke responses within the CIC for each electrode diameter with a single stimulation pulse, although the number of cells that reached stimulation, the threshold to evoke spikes, and the number of evoked spikes varied with electrode diameter. While we could evoke stimulation with the 10 μm diameter electrodes in some cells, we noticed that fewer cells responded to stimulation with 10 μm diameter electrodes than at other diameters.

To explore this observation further, we used a subset of cells which were approximately equidistant to all three sizes of electrodes. This allowed us to compare responses to different size electrodes within the same cell. In cases where electrodes were not exactly equidistant, we minimized the distance between the cell of interest and the smaller electrodes. This resulted in a mean distance to the electrode of 31.8, 43.3, and 80.0 μm for 10, 20, and 30 μm diameter electrodes, respectively, for this within cell electrode comparison. In 53% of the cells (*n* = 21/40), we failed to get stimulation from a 10 μm diameter stimulating electrode in a cell that responded to a 20 or 30 μm diameter electrode that was equidistant or farther from the 10 μm diameter electrode, indicating that 10 μm diameter electrode are less effective at recruiting RGC responses than larger diameters. While, it may be expected that smaller electrodes could be less effective at stimulating at longer distance, within this restricted range of the within cell comparison, we found no correlation between distance from cell and likelihood of stimulation.

**Figure 6.**
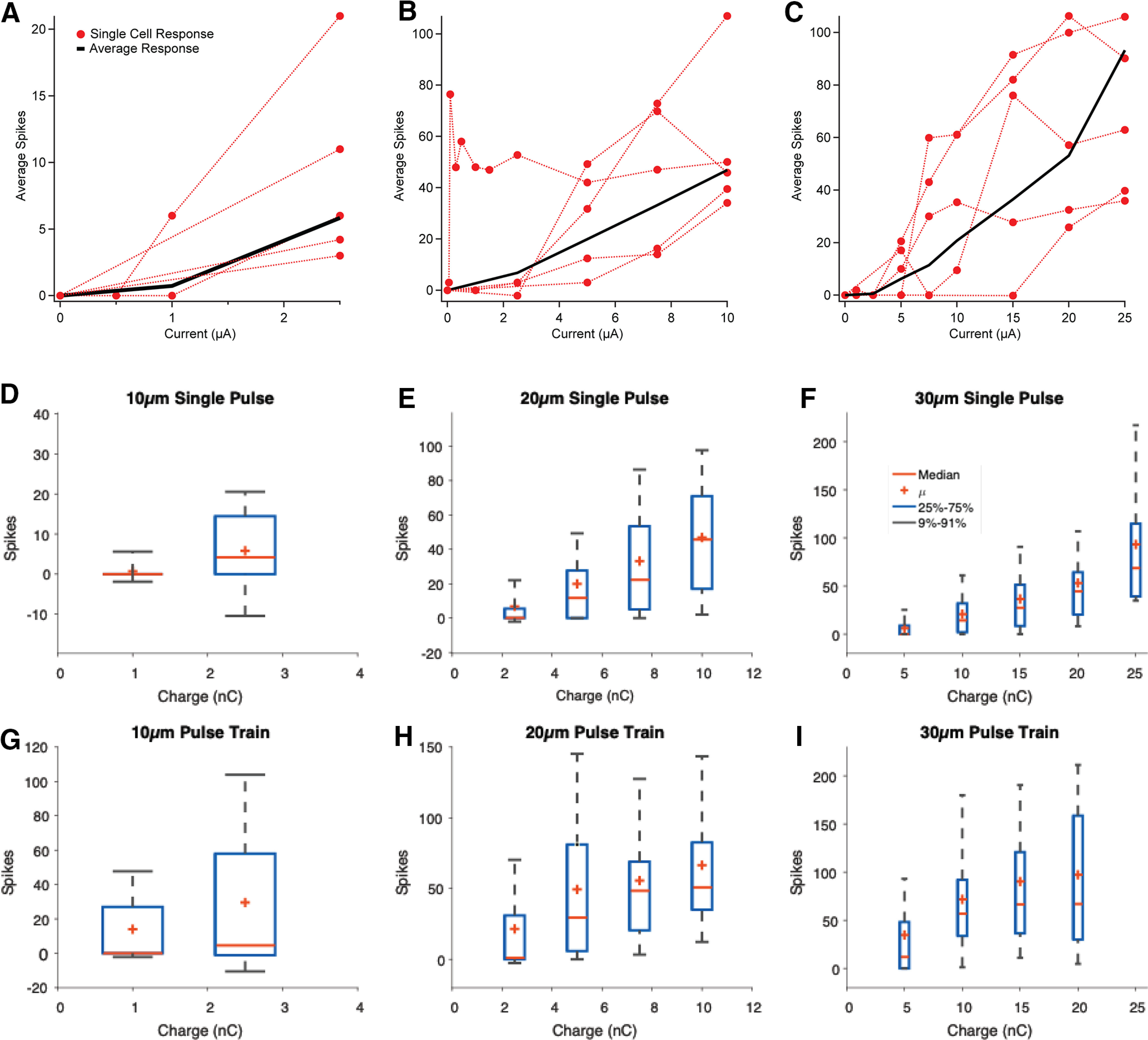
RGC stimulation with 1 ms pulses for 10, 20, and 30 μm electrodes. ***A–C***, Stimulus response relationship for five individual example RGCs (red) and average response (black) within the CIC limits of each respective electrode size. ***C–F***, Stimulation thresholds for stimulation at each electrode diameter for single pulses and (***G–I***) pulses trains. Red plus denotes mean.

For all electrode diameters, we found that spike number generally increased monotonically with increasing current for all diameters when averaged across a group of cells, although there was significant cell to cell heterogeneity of evoked responses (Fig. 6). At equivalent stimulating charge, smaller electrodes evoke a greater spiking response. For example, at 5 and 10 nC charge for a single stimulus pulse, a 20 μm diameter electrodes evoked 3.1× (*t* test, *p* < 0.01) and 2.4× (*t* test, *p* < 0.01) more spikes than a 30 μm diameter electrode, respectively (Fig. 6*D*,*E*). These observations also hold true for pulse train stimulation, although more spikes were evoked with pulse trains than single pulse stimulation across all electrode diameters and stimulation charge (Fig. 6*F–H*).

**Figure 7.**
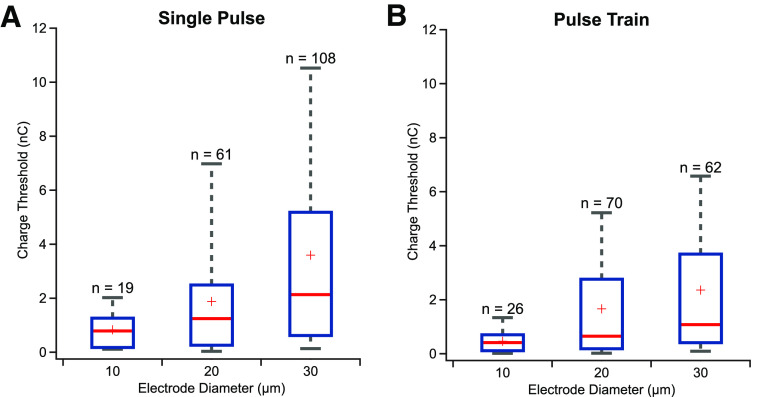
Comparison of 10, 20, and 30 μm responses: (***A***) charge threshold for stimulation for single pulses and (***B***) pulse trains. Blue box denotes 25%–75% range. Gray whiskers denotes 9%–91% range. Red line denotes median, and red plus denotes mean.

A key measure used to describe the efficiency of retinal stimulation is charge threshold, defined as the charge level needed to evoke at least one spike on 50% of the trials. Here, we measured the charge threshold for our different electrode diameters and found that stimulation thresholds increased with electrode size. The threshold charge was 0.83, 1.8, and 3.6 nC for 10 μm (*n* = 19), 20 μm (*n* = 61), and 30 μm (*n* =108) diameter electrodes, respectively, using single stimulus pulses (Fig. 7*A*). Results for pulse train stimulation mirrored the findings for single pulse stimulation with the exception that pulse train stimuli had lower charge thresholds than single pulses (unpaired *t* test: *p* < 0.05). For pulse trains, the charge thresholds were 0.46, 1.6, and 2.4 nC for 10 μm (*n* = 26), 20 μm (*n* = 70), and 30 μm (*n* = 62) diameter, respectively (Fig. 7*A*). Taken together, these results indicate that smaller electrodes are generally more efficient at evoking RGC spiking, on a per charge basis, with the important caveat, that 10 μm diameter electrodes evoked responses in fewer cells compared with 20 and 30 μm diameter electrodes.

While numerous studies have examined stimulation threshold in the past, the absolute range of responses that can be evoked within the usable range of electrical charge has been considered far less. This is an important property to consider for prosthetic stimulation as it ultimately determines the range of visual information that can be encoded and the resolution of continuously varying responses that can be discriminated. If we consider a stimulus that only produces threshold responses (a single spike 50% of the time), this would probably not reproduce satisfactory vision as it would be a very weak percept to encode a range of input conditions.

**Figure 8.**
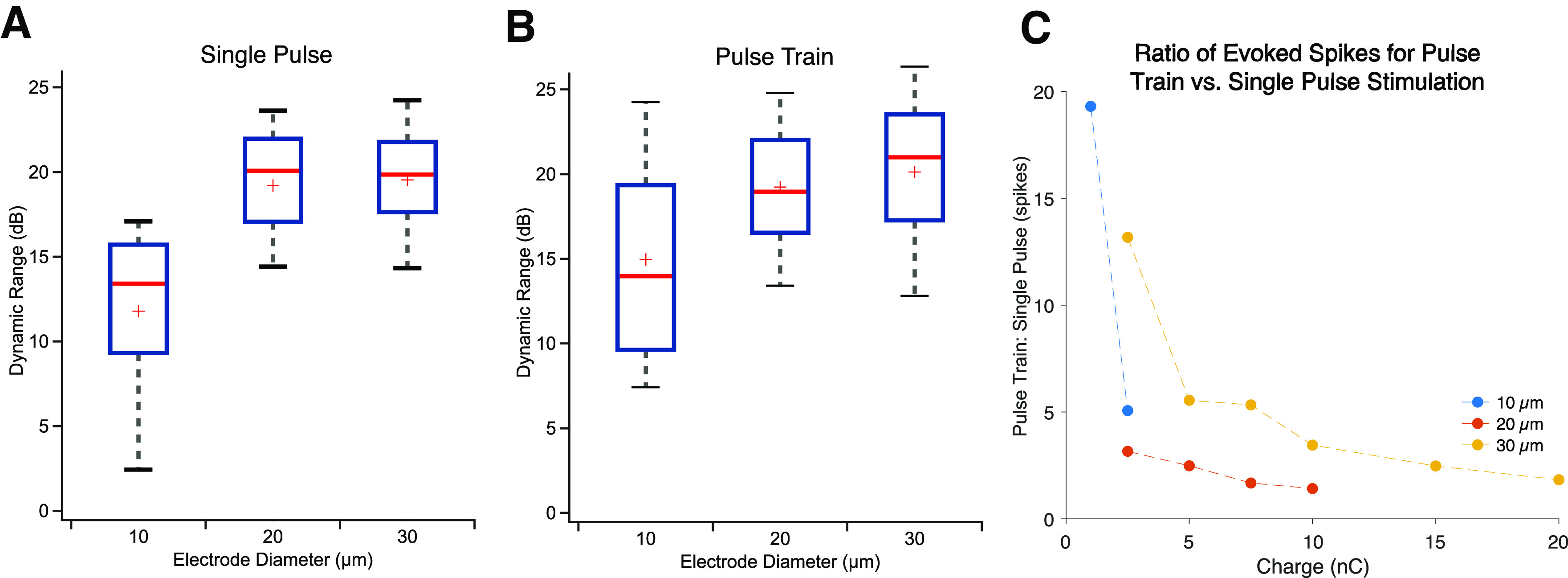
Comparison of available dynamic range of responses for 10, 20, and 30 μm diameter electrodes for (***A***) single pulses, and (***B***) pulse trains, expressed in decibels. Blue box denotes 25%–75% range. Gray whiskers denotes 9%–91% range. Red line denotes median, and red plus denotes mean. ***C***, Comparison of retinal stimulation efficacy with pulse trains versus a single pulse stimulus. Ratio of spikes evoked for pulse train to single pulse stimuli at equivalent stimulating charge levels for each stimulating electrode diameter.

Therefore, we quantified the dynamic range as the ratio of the maximal number of spikes evoked within the CIC limit and the spikes evoked at the threshold (Fig. 8*A*,*B*). Consistent with our observation that larger electrodes can evoke more spikes within their CIC limit, we found the dynamic range of RGC responses to subretinal stimulation differed over electrode diameter (one-way ANOVA, *p* < 0.05), and the dynamic range for 10 μm diameter electrodes was significantly different compared with compared with 20 or 30 μm electrodes for both single stimuli and pulse trains. The dynamic range for a 30 μm electrode was 1.6× and 1.3× that of 10 μm diameter electrodes on the decibel scale, for single and pulse trains stimulation, respectively.

What could account for these differences in stimulation between electrode sizes? Electrical stimulation of neural tissue occurs when injected current creates an electric field in the extracellular space that encompasses all or part of the neuron. The electric field causes a redistribution of charge along the cell surface, altering transmembrane voltage and leading to the activation of voltage-gated ion channels that trigger an action potential or synaptic release. The magnitude of the electric field is proportional to the stimulating charge density. Consequently, charge density has been discussed as a metric to normalize the amount of charge needed for stimulation across different electrode geometries (Palanker et al., 2005), however, when comparing previous studies with varying electrode sizes there is a wide range of results reported for the required charge density for effective stimulation (Stett et al., 2000; Tsai et al., 2009; Yang et al., 2011; Im and Fried, 2015; Corna et al., 2018). Importantly, Corna et al. (2018) demonstrated that stimulation threshold expressed in charge density is not conserved for variety of total electrode areas, when simulating large electrodes by grouping 30 μm diameter electrodes. For epi-retinal stimulation, Sekirnjak et al. (2006) also found charge density was not conserved across a range of electrode areas.

**Figure 9.**
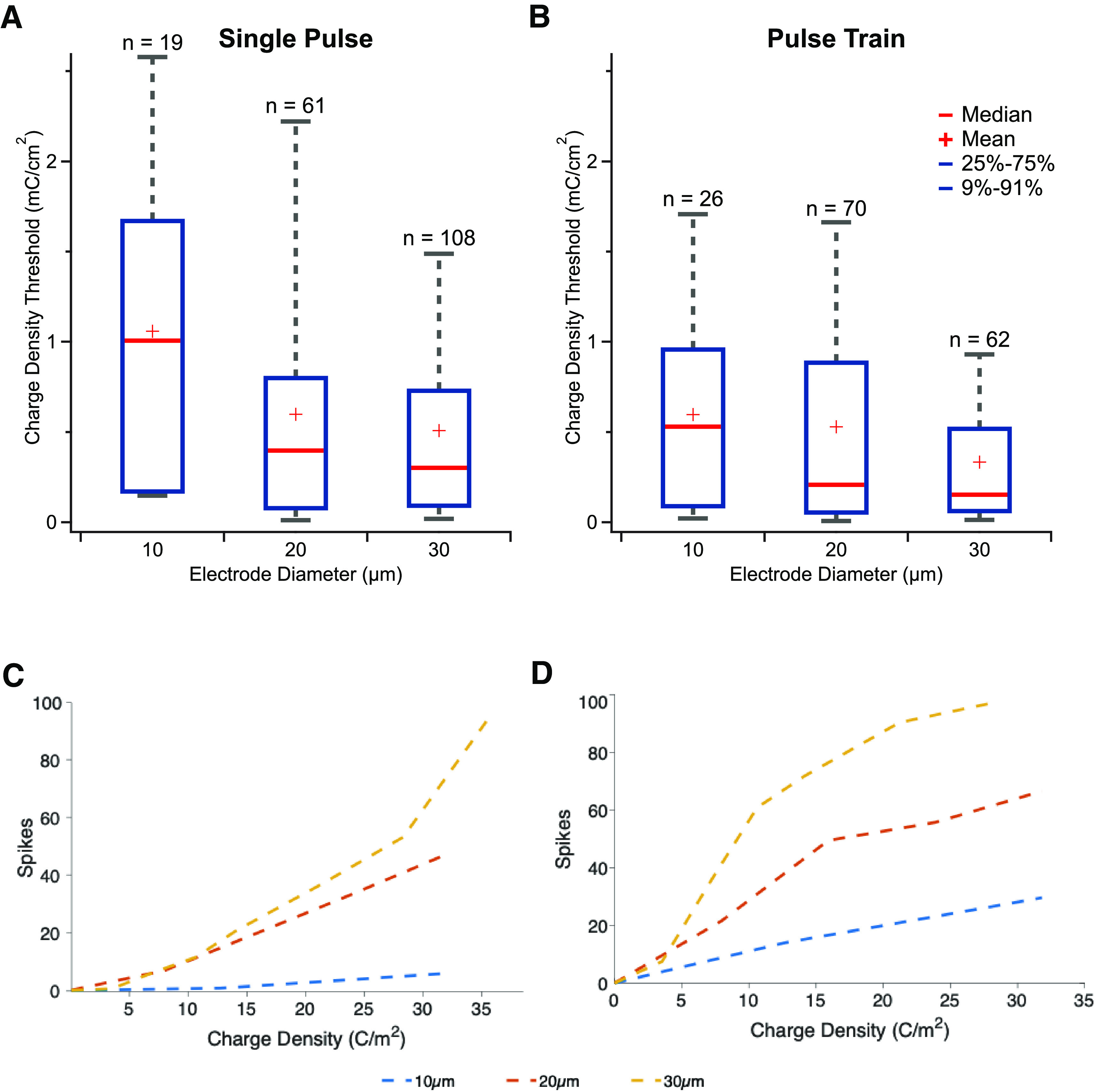
Charge density thresholds for 10, 20, and 30 μm electrodes. ***A***, Charge density thresholds for single pulses (***B***) and pulse trains. ***C***, Intensity response relationship for evoked spikes versus stimulating charge density for single pulses (***D***) and pulse trains.

To examine this question for subretinal stimulation at relevant electrode diameters, we converted charge thresholds to charge density thresholds based on the geometric area (Fig. 9*A*,*B*). Charge thresholds significantly differed across electrode diameters (one-way ANOVA, *p* < 0.05). In contrast to charge thresholds, we observed the highest charge density thresholds for the smallest electrodes and a decreasing trend in charge density thresholds with increasing electrode area, for both single stimuli and pulse trains, with a significant interaction between 10 and 30 μm diameter electrodes.

As shown above, threshold is not the only difference between electrode diameters; the absolute range of responses evoked over a range of stimulation currents is another important metric that differs between electrode sizes. To understand how charge density might contribute to the differences in stimulation efficiency across a range of stimulation levels, we plotted spiking response against charge density for our different electrode diameters (Fig. 9*C*,*D*). At equivalent charge density, the spiking response measured for RGCs differs strongly between electrode diameters. Larger diameter electrodes are more effective at eliciting spikes for both single pulses and pulse trains, even when the charge density is equal. These data suggest that stimulation efficacy is not well explained by charge density, even for electrode geometries that are relatively similar, such as our planar electrodes ranging from 10 to 30 μm diameter. Specifically, this allows us to reject the hypothesis that charge density alone determines neural stimulation over a relevant range of electrode areas.

What could account for differing stimulation efficiencies at the same charge density and electric field strength? Given that electric field strength is non-uniform and decays with distance from the electrode, the location of a neuron within the electric field is an important consideration in determining the efficacy of stimulation, particularly for electrodes on the scale of individual neurons. We hypothesized that differences in the size of the electric field evoked by different size electrodes may account for differences in stimulation efficiency with electrode size, even when charge density is constant.

**Figure 10.**
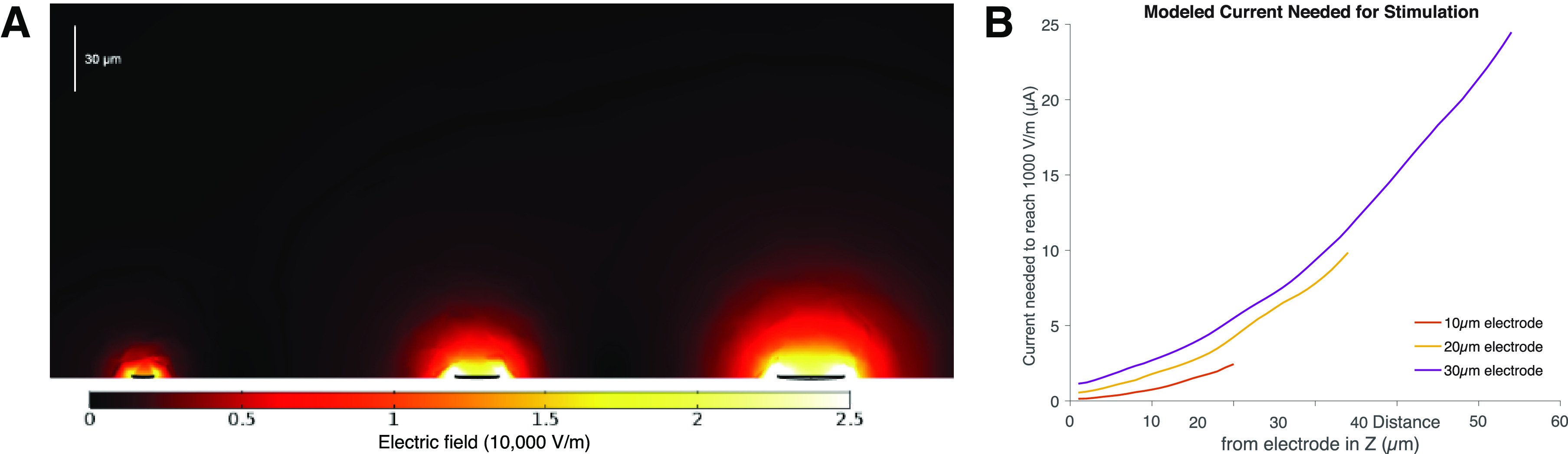
Modeling penetration depth of stimulating electric field for different iridium oxide electrode diameters. ***A***, Cross section (*Y-Z* plane) heatmap visualization of the electric field induced by the injection of current from a 10, 20, and 30 μm (left to right) electrode at charge injection limits for a 1 ms pulse in a 3-D finite element model of retinal tissue. The penetration depth and magnitude of electric field are directly related to the size of the electrode and injected current. ***B***, Calculation of the stimulation current needed to induce a 1000 V/m electric field at a *z*-height above the stimulating electrode. The required current curves for each diameter terminate at the respective maximum current for a 1 ms pulse at the charge injection limit.

To test this idea, we modeled the electric field using a 3-D finite element model for each electrode diameter. For a fixed current density corresponding to the maximum injectable charge at each electrode diameter in a 1 ms pulse width and assuming 1000 V/m as a minimum electric field to induce stimulation (Moghadam et al., 2013). Visualizing the magnitude and spread of the electric field in the y and z dimensions reveals that the spread of the stimulating electric field depends strongly on electrode size (Fig. 10*A*). Within the limits of the electrode CIC, the effective stimulation depth of a 10 μm diameter electrode for a 1 ms pulse width and 2.5 μA stimulation current is limited to a ∼20 μm radius from the surface of the electrode. The modeled stimulation depth of 20 and 30 μm electrodes extends much further to a 35 μm and 55 μm radius, respectively. The short depth of effective stimulation may account for the poor reliability of retinal stimulation with 10 μm (47%) electrodes versus 20 and 30 μm electrodes (100%) and supports our hypothesis that the difference in the stimulation efficacy between electrode sizes can be attributed to the penetration depth and/or spread of the electric field. The electric field shape over the scale of target neurons provides a framework for understanding differences in stimulation efficiency between different electrodes sizes when current density is held constant. Importantly, the penetration depth of electric field within the extent of safe injectable charge limits suggests a significant limitation on electrode scaling for subretinal prostheses (Fig. 10*B*).

## Discussion

Development of high-acuity retinal prosthetics may enable treatment of common forms of blindness caused by retinal degeneration. Advances in microfabrication and the development of high CIC electrode materials have enabled the development of high-resolution (>20/400 acuity) retinal prosthetics; however, there remains a poor understanding of the constraints placed on neural stimulation by electrodes at this small scale. Here, we characterized the neural responses to high density retinal prosthetic stimulation electrodes made of sputtered iridium oxide (SIROF) with 10, 20, and 30 μm diameter. Importantly, we first characterized the charge injection limits to only examine responses within their electrochemical capabilities. Here, we identified key aspects of neural stimulation that are constrained by these electrochemical limitations.

Electrical stimulation of retina has been studied over many decades. Much of the fundamental electrophysiological characterization of retinal responses to intracellular or extracellular stimulation has been done using glass micro-pipettes embedded within the retina to deliver charge. While this can create highly focal electric fields, the experimental parameter space is not bounded by practical limits on current, charge, geometry, or electrode material. Other studies have used more realistic geometry with planar metal electrodes (Stett et al., 2000, 2007; Yang et al., 2011; Cai et al., 2013; Sim et al., 2014; Im and Fried, 2015; Stutzki et al., 2016; Ho et al., 2019). While, many of these studies characterized stimulation efficacy in healthy retina with an intact photoreceptor cell layer, which significantly affects stimulation thresholds (Stett et al., 2000, 2007; Boinagrov et al., 2014; Sim et al., 2014). Few studies have systematically explored the range of responses that can be evoked above stimulation threshold or the relationship to electrode size/geometry. In addition, there have been extensive theoretical (Palanker et al., 2005) and experimental efforts (for review, see Sekirnjak et al., 2006; Corna et al., 2018), however, as these reviews of experimental results reveal there is considerable variability in the published results, leaving much uncertainty about the specific physical considerations that drive stimulation efficacy. Importantly, charge injection limits have rarely been considered beyond a brief mention of the reported CSC for the electrode material. To the best of our knowledge, this is the first report of dynamic range for electrodes of this scale within the specific charge injection limits of SIROF electrodes.

Working within the CIC limits of SIROF electrodes of different sizes, we evaluated the effectiveness of stimulation at each electrode size in terms of stimulation thresholds and the dynamic range of stimulation. Importantly, we found that stimulation thresholds occur at 38% (10 μm), 23% (20 μm), and 18% (30 μm) of the maximum available charge within the CIC. Although the thresholds were within the CIC for all diameters, maximal responses were limited by the CIC of the electrode rather than the physiology and indicate that the dynamic range of responses is strongly influenced by electrode size, a novel finding that has not been appreciated by previous studies focused on stimulation thresholds. Importantly, of those cells that were responsive to stimulation with a 30 μm electrode, <50% could be stimulated with a 10 μm electrode that was as close or close to the cell, indicating that 10 μm diameter electrodes are less reliable at evoking responses than larger electrodes. These limitations on dynamic range and efficiency have important implications for retinal prosthetic designs.

In addition, this work also revealed how current magnitude and total charge relate to stimulation efficiency and dynamic range of responses. We found that the absolute current magnitude of the delivered charge has a significant influence on retinal stimulation efficacy. Past work has often focused on total charge delivered or charge density. In contrast, we show here that neither of these properties fully account for stimulation efficiency. We find that shorter duration, higher current magnitude stimulation parameters evoked more spikes than longer pulses of a lower current magnitude.

Past studies of subretinal stimulation have identified that short latency responses (<5–10 ms) are characteristic of direct RGC stimulation and longer latency responses (>10 ms) can be attributed to indirect network mediated stimulation (Jensen et al., 2005; Sekirnjak et al., 2006; Margalit et al., 2011; Sim et al., 2014). Notably, some previous studies have proposed that longer pulse widths yield greater selectivity for indirect stimulation (Margalit et al., 2011; Boinagrov et al., 2014). Although we have not attempted to assign any functional designation to the different spike latencies here, our results indicate that this must hold true only at a fixed current level where a longer pulse duration results in more delivered charge. Here, we show that greater current is more effective at evoking both short and long latency spikes for an equivalent charge.

These findings have important implications for the design of high acuity retinal prostheses. Charge density is often considered as the relevant parameter for comparing neural stimulation across different electrode scales. Here, we find that larger electrodes, with a capacity to deliver greater current, are more effective at evoking RGC responses than smaller electrodes, even at the same current density. These results consistent with recent work by Corna et al. (2018), where they found that larger electrode areas used by grouping multiple electrodes together had lower charge density thresholds and were more effective at evoking spikes at equivalent charge density. This indicates that when designing hardware to deliver current, it is important to consider the maximum range of spikes that can be evoked based on the limits of the current source. For retinal implants that can only source a limited or fixed current output at each electrode, longer pulse widths may be required. In contrast, for implants that can supply a wider current range, a short pulse width and current injection near the limit of the CIC may achieve the broadest range of stimulation responses and is more efficient, yielding the most spikes for a given amount of charge. Given, that 10 μm diameter electrodes failed to evoke responses in a majority of ganglion cells that responded to larger diameter electrodes, there may be a practical limit for miniaturizing the scale of planar iridium oxide electrodes used for effective retinal stimulation. Our results from electric field modeling indicate that this limit is related to the size of the electric field relative to target neurons. Consequently, this limitation on minimum electrode size also limits the possible spatial resolution of an implanted electrode array.

The limited dynamic range of RGC responses within the safe injectable charge limits at all diameters indicates a need for better electrode materials at small electrodes scales. In general, the number of evoked spikes increases linearly with increasing current and does not saturate within the limits of CIC for the electrode sizes tested here. Alternative electrode materials such as conductive polymers including poly 3,4-ethylenedioxythiophene (PEDOT) or nanostructured platinum or iridium may enable higher charge injection and improve the efficacy of stimulation at 10 μm electrode size or smaller (Ganji et al., 2017, 2019). Additionally, high acuity retinal prostheses may require 3-D electrodes that penetrate through the retina to reduce electrode-tissue separation and thereby improve the effectiveness of stimulation at small electrode scales (Ganesan et al., 2010; Flores et al., 2018).

There are several caveats to consider with regards to this experimental approach and scope of our results. The stimulus pulse duration did not extend past 5 ms because of hardware restrictions of the current source. Longer pulse durations extending up to 50 ms and proportionally lower current may yield further insights into the strength-duration characteristics of retinal stimulation within CIC limits, though our results here suggest that longer pulses are less efficient on a per charge basis. Also, the stimuli tested here consist of single pulses or 20× pulse trains at 500 Hz. While using pulse trains may be an effective strategy to reduce stimulation threshold and increase the dynamic range of spikes, the responses began to saturate at the maximum charge injection limit, indicating a diminishing impact of repetitive stimulation. Further characterization using subretinal stimuli at varying frequency and duty cycle may yield greater insight into the possible range of evoked responses that can encode functional visual information within CIC limits.

Throughout this work, we have averaged across a heterogeneous population of ganglion cell types. Indeed, our data showed a diversity of the range and latency of spiking responses, a relatively broad distribution of current thresholds, and different current/spiking functions. We feel justified in using the mean response of the population, because currently there is insufficient data to suggest the possibility of selective targeting of any particular population of ganglion cells (but see Fan et al., 2019). Therefore, a general goal of retinal prosthetic stimulation is to recruit activity in the largest population of ganglion cells. For an actual prosthesis, the retina receives concurrent stimulation from an array of electrodes resulting in a complex pattern of evoked responses across diverse cell types. Although we have characterized responses only from a single cell using a single stimulating electrode, this a necessary foundational step to determining the minimum size at which each electrode is independently effective within a dense array.

Ultimately, the relationship between injected charge and neural stimulation results from complex interactions that depend on the shape and magnitude of the electric field, the geometry of neural processes traversing the electric field, and the complement of voltage-gated ion channels in the target neurons. Consequently, a universally scalable relationship between the magnitude of charge delivered through stimulating electrodes and the resulting effectiveness of retinal stimulation will be complex, however, this approach of characterizing evoked responses within quantified CIC limits can help establish a framework to compare between or develop new microelectrodes for high visual acuity subretinal prosthesis.

## Synthesis

Reviewing Editor: Miriam Goodman, Stanford University Decisions are customarily a result of the Reviewing Editor and the peer reviewers coming together and discussing their recommendations until a consensus is reached. When revisions are invited, a fact-based synthesis statement explaining their decision and outlining what is needed to prepare a revision will be listed below. The following reviewer(s) agreed to reveal their identity: Pietro Artoni. Note: If this manuscript was transferred from JNeurosci and a decision was made to accept the manuscript without peer review, a brief statement to this effect will instead be what is listed below.

Thank you for your attention to reviewer feedback. Both reviewers were largely satisfied with the revised manuscript. Rev2 asks that you define ‘efficacy’ for the purposes of this study - a recommendation that I endorse. The definition could be explicitly provided upon the first use of the phrase of ‘stimulation efficacy’, for example.

Rev1

The authors answered my concerns. They have explained the experiment much more in details in the methods section.

Rev2

The manuscript demonstrates for the first time stimulation of ganglion cell activity using 10 μm diameter electrodes within safety limits. Secondly, the manuscript shows that for subretinal configuration, small electrodes (with global return electrode) provide a limited dynamic range (i.e. firing rate) as compared to larger electrodes (30 μm). This result is novel.

The authors use the word “efficacy” twelve times without providing a definition in the manuscript. They explain “efficacy” in the rebuttal letter as “the ability to produce the desired effect / i.e. the ability of electrical stimulation to produce ganglion cell spiking within the practical limits of electrical charge”. This definition may be understood as the ability to produce one ganglion cell spike per stimulus or the ability to produce many ganglion cell spikes (i.e. to increase) with increasing amplitude and thereby offering a high dynamic range. Apparently the authors favor the second option. But than, it remains somewhat unclear why they decide to estimate threshold as the minimum stimulus which evokes “one spike on 50% of trials”. The argument that others have used this definition before is controversial, since previous work may have targeted a different “efficacy”. In short, some prosthetic approaches (pioneered by EJ Chichilnisky’s Lab) aim for one spike / stimulus. In this case “efficacy” is defined as a binary event and consequently threshold is defined as “one spike on 50% of trials”. In subretinal studies “efficacy” is usually understood as increasing the firing rate of ganglion cells. But than, why not linking stimulation threshold to the firing rate ?

There are few aspects, which would improve the manuscript.

Was the recording temperature controlled in the experiment and if so, what was the value ?

In Figure 5 the spiking pattern to various stimuli is shown as a heatmap, without spiking prior to ethe stimulus and little spiking between 100 - 500 ms post-stimulus. Usually, photoreceptor-degenerated mouse retinae show a high spontaneous activity (hyperactivity, see SF Stasheff’ s work). This phenomenon seem to be missing here. Any comment ?

Two remarks, just for the author’s information:

Schoen & Fromherz (2008) published an analytical equation for the voltage above stimulation electrodes of different sizes (eq. A1 in Schoen& Fromherz) and not only for one electrode size, as the authors mention. This equation could have been compared to the author’s COMSOL simulation.

The threshold charge density reported by the authors for single pulses and 30 μm electrodes (∼ 0.5 mC/cm2) corresponds very well to the threshold reported by Corna et al (2018) for the same electrode size, electrode material and animal species. This is remarkable.
